# An Overview of miRNAs Involved in PASMC Phenotypic Switching in Pulmonary Hypertension

**DOI:** 10.1155/2021/5765029

**Published:** 2021-10-07

**Authors:** Weifang Zhang, Zeying Tao, Fei Xu, Qian Diao, Juan Li, Lu Zhou, Yaxin Miao, Shanshan Xie, Jinjin Wan, Ruilai Xu

**Affiliations:** ^1^Department of Pharmacy, The Second Affiliated Hospital of Nanchang University, 330006 Nanchang, Jiangxi, China; ^2^Department of Pharmacology, School of Pharmaceutical Science, Nanchang University, Nanchang, Jiangxi 330006, China; ^3^Medical College of Nanchang University, Nanchang, Jiangxi 330031, China

## Abstract

Pulmonary hypertension (PH) is occult, with no distinctive clinical manifestations and a poor prognosis. Pulmonary vascular remodelling is an important pathological feature in which pulmonary artery smooth muscle cells (PASMCs) phenotypic switching plays a crucial role. MicroRNAs (miRNAs) are a class of evolutionarily highly conserved single-stranded small noncoding RNAs. An increasing number of studies have shown that miRNAs play an important role in the occurrence and development of PH by regulating PASMCs phenotypic switching, which is expected to be a potential target for the prevention and treatment of PH. miRNAs such as miR-221, miR-15b, miR-96, miR-24, miR-23a, miR-9, miR-214, and miR-20a can promote PASMCs phenotypic switching, while such as miR-21, miR-132, miR-449, miR-206, miR-124, miR-30c, miR-140, and the miR-17~92 cluster can inhibit it. The article reviews the research progress on growth factor-related miRNAs and hypoxia-related miRNAs that mediate PASMCs phenotypic switching in PH.

## 1. Introduction

Pulmonary hypertension (PH) is a serious cardiopulmonary disease that occurs as a primary rare disease or as a concurrent condition of various cardiac, pulmonary, or systemic diseases. PH has multiple predisposing factors, but all forms of PH show a common arteriopathy, including pulmonary vasoconstriction, vascular remodelling, and subsequent vascular lumen occlusion, although their evolution and prognosis vary depending on the aetiology. These alterations then trigger an increase in pulmonary vascular resistance and compensatory right ventricular hypertrophy, which ultimately result in mortality [1].

Pulmonary vascular remodelling involving the intima, media, and adventitia is a critical pathological change in all PH types. In the process of pulmonary vascular remodelling in patients with PH, vascular endothelial injury, vascular media hypertrophy, muscle fibrosis of peripheral vessels, and an increase in extracellular matrix (ECM) often occur, resulting in conformational changes. As a result, the pulmonary vascular lumen will constrict, small resistant pulmonary arteries will be progressively occluded, and angioproliferative plexiform lesions will form, which regulate PH progression [2].

The overproliferation of pulmonary arterial smooth muscle cells (PASMCs), an important component of the vascular media, caused by the disruption of the proliferation/apoptosis balance of PASMCs and phenotypic switching is the main cause of pulmonary vascular remodelling in PH. SMCs can contract blood vessels and regulate vascular tension, blood pressure, and blood flow distribution. Under normal conditions, they are static and differentiated, showing low proliferation and low synthetic activity. However, under pathological conditions such as hypoxia and inflammation, SMCs undergo phenotypic switching, which is characterized by hyperplastic and antiapoptotic properties. Because of the overproliferation and migration of synthetic phenotypic SMCs in a dedifferentiated state and the secretion of collagen, elastin, proteoglycan, and ECM contractile proteins, pulmonary arterioles and capillary walls become thickened or even occluded, the lumen of blood vessels becomes narrowed, and blood flow resistance increases; these changes, in turn, increase the pressure in the pulmonary arteries and promote the development of pulmonary vascular remodelling [3]. Therefore, PASMCs phenotypic switching is a key link in pulmonary vascular remodelling and is particularly important in PH. The mechanism behind phenotypic switching is complicated. Many studies have investigated the mechanism of PASMCs phenotypic switching, and some have involved various signalling pathways, such as the MAPK/ERK1/2 and PI3K/AKT signalling pathways [4, 5]. Interventions targeting the abnormal differentiation, migration, and proliferation mechanisms of PASMCs have been shown to effectively inhibit pulmonary vascular remodelling and treat PH.

MicroRNAs (miRNAs) are 18–22 nucleotides (nt) in length and are single-stranded noncoding small RNAs. Binding to the 3′-untranslated region (3′-UTR) of messenger RNAs (mRNAs) to degrade mRNA and/or inhibit target gene translation is the primary mode of action of miRNAs, which widely regulate gene expression. The discovery of miRNAs and their function constitutes a major breakthrough in the field of medicine. The role of miRNAs in various cardiovascular diseases, including ischaemia, tumour angiogenesis, and atherosclerosis (AS), has attracted considerable attention in recent decades. In particular, the abnormal expression of miRNAs in PH has attracted much recent attention among scholars. miRNAs are involved in the differentiation of vascular endothelial cells and PASMCs. Under the influence of inflammation, hypoxia, external stimuli, and other factors, miRNAs can regulate the production of cytokines, chemokines, and various growth factors by regulating the expression of related genes. These modulations further alter the biological behaviour of vascular endothelial damage, SMC proliferation, migration and phenotypic switching, and abnormal ECM deposition, which are the cellular and molecular bases of PH [6].

Despite recent progress in our understanding of the pathophysiological mechanism of PH and significant improvements in symptomatic treatment, the rapid progression and lethal course of the disease have not substantially changed [7]. There is therefore an urgent need to identify new potential therapeutic targets. The treatment of PH should not only solve the problem of vasoconstriction but also address the deeper problem of vascular remodelling. Controlling the expression of genes and proteins can fundamentally regulate the occurrence and development of PH. miRNA dysregulation is closely related to the physiopathology of PH. Consequently, miRNA-based therapeutics have constituted a new hope for the reversal of the PH process in clinical practice. In view of the key regulatory role of miRNAs in PASMCs phenotypic switching, this review describes the current research progress regarding miRNAs involved in PASMCs phenotypic switching in PH from two aspects—growth factor-related miRNAs and hypoxia-related miRNAs—to further clarify the pathogenesis of PH and provides an important experimental and theoretical basis for the application of miRNAs in targeted PH therapies.

## 2. Brief Description of PH, Phenotypic Switching, and their Relationship

### 2.1. Pulmonary Hypertension

#### 2.1.1. Overview of PH

PH refers to a class of progressive diseases of different aetiologies. The main cause of the disease is primary pulmonary arteriolar lesions leading to increased pulmonary artery resistance and eventually to death from right heart failure [8]. PH was divided into five categories at the sixth World Symposium on Pulmonary Hypertension (WSPH): pulmonary arterial hypertension (PAH), PH associated with left heart disease, PH associated with lung disease/hypoxia, PH due to pulmonary arterial obstructions, and PH with unclear and/or multifactorial mechanisms [9]. By 2011, in all its variant presentations, PH was estimated to affect up to 100 million people worldwide [10].

Since 1973, PH has been defined as a mean pulmonary arterial pressure (mPAP) ≥ 25 mmHg; however, the definition was recommended to be changed to mPAP > 20 mmHg at the sixth WSPH [11]. .PH has previously been called an orphan disease, that is, a condition that affects relatively few individuals and is overlooked by the medical profession and pharmaceutical industry [12]. Today, PH is no longer ignored, and research on PH is intensifying. Important findings have greatly improved our understanding of this disease and have helped guide patient management. In 1891, Dr. Romberg, a famous German doctor, reported the first PH case, describing a 24-year-old patient who experienced severe dyspnoea, chronic drowsiness, and cyanosis prior to death. Romberg's autopsy report of the patient revealed vascular lesions in the small pulmonary arteries and severe right ventricular hypertrophy. Romberg, however, was unable to identify a pathological cause of the pulmonary artery lesions and ultimately described them as “pulmonary vascular sclerosis” of unknown origin [13, 14]. In the 1940s, Coumard used cardiac catheters to directly measure pulmonary artery pressure, and people began to understand PH from a haemodynamic perspective. Thereafter, Dresdale reported a patient with unexplained PH and termed it primary pulmonary hypertension (PPH). In the 1960s, aminorex caused a PH epidemic in Europe, which attracted the attention of the European medical community and even the World Health Organization (WHO). Prompted by the aminorex incident, the WHO held its first conference in Geneva in 1973 to establish an expert group on PH to define its aetiology and develop a pathological nomenclature for PH. The team of more than a dozen authoritative experts in Europe and the United States divided PH into two categories: PPH and secondary PH. Since then, the National Institutes of Health National Heart, Lung, and Blood Institute launched a nationwide multicentre PPH registration study. At the 2nd World PH Conference in 1998, PH was divided into five clinical diagnostic categories; although they are updated every year, these five classification principles are maintained [14].

Group I PAH is the most important among the categories due to its aggressive nature, poor survival outcome, and limited treatment options. With efforts over the last three decades, the survival of patients with group I PAH has improved but is still suboptimal, and further improvement remains an unmet challenge. PAH is a dangerous disease that is nonspecific, has a poor prognosis, and lacks an effective treatment. The Registry to Evaluate Early and Long-term PAH Disease Management (REVEAL) study showed a five-year survival rate of 57% from the time of diagnostic right heart catheterization (RHC) [15]. Over the past two decades, the long-term survival of patients with PAH has markedly improved. The current average survival time of PH patients is 6 years, compared to 2.8 years in the 1980s. Similarly, the annual survival rate of PAH patients ranges from 86% to 90%, up from 65% in the 1990s. Despite these improvements, PAH still imposes a massive clinical and economic burden. While the number of PAH-related hospitalizations declined between 2001 and 2012, the average cost and length of PAH-related treatment increased, while the inpatient mortality rate did not significantly decrease and life expectancy remains low [16–18].

Because of the nonspecificity of the early symptoms, most PAH patients often delay diagnosis. The condition then worsens and finally enters the irreversible stage, where treatment is difficult and the prognosis is poor. The early diagnosis and evaluation of PAH are essential for guiding the treatment, improving prognosis, and improving the survival and quality of life of patients with PAH. Methods for the evaluation and detection of PAH have rapidly progressed in recent years. The commonly used diagnosis and treatment methods are the six-minute walk test, cardiopulmonary exercise testing, lung function testing, chest X-ray, electrocardiography, ultrasonic cardiography, chest computed tomography (CT) and CT pulmonary angiography, lung ventilation/perfusion single photon emission CT, magnetic resonance imaging, and RHC. RHC is a traumatic and invasive examination, and the procedure is complex, difficult to repeat, and has certain risks; however, it allows the direct acquisition of accurate and reliable haemodynamic data and excludes intracardiac shunts, abnormal drainage, and other serious left heart diseases to help identify the cause of PAH and test the responsiveness to therapeutic drugs. Thus, RHC remains the gold standard diagnostic method for PAH [19, 20].

Regarding treatment, in addition to the traditional comprehensive treatments of oxygen inhalation, cardiotonic agents, diuretics, anticoagulants, and vasodilators (whose effect is not favourable in patients with a negative acute pulmonary vascular response upon testing), the application of targeted drugs has brought hope for improving the quality of life and prolonging the survival of patients with advanced PH. Before 1995, clinicians used traditional medicines such as digitalis, diuretics, and potassium supplements and antihypertensive drugs to treat PH, but patient prognosis was poor, and the mortality rate was high. As research in PH mechanisms has progressed, targeted drugs with different modes of action have entered the market, and PH is currently treated via the oral, inhalation, subcutaneous injection, and intravenous drip routes. The prognosis of PAH patients has thus gradually improved; the 1-, 3-, and 5-year survival rates of patients have increased from 68%, 48%, and 34% to 86%, 69%, and 61%, respectively [21, 22]. Currently, there are three main traditional categories of targeted therapeutic drugs: endothelin receptor antagonists (bosentan and ambrisentan), phosphodiesterase 5 inhibitors (sildenafil, tadalafil, and vardenafil) and prostacyclin (epoprostenol, iloprost, triprostanil, and beraprost). In addition, some novel PAH-targeted therapeutic drugs, such as the soluble guanylate cyclase activator Adempas and the prostacyclin receptor agonist Uptravi, have shown promising results.

#### 2.1.2. Pathophysiological Characteristics of PH

In normal pulmonary vessels, the pulmonary arteries are the main component of the pulmonary vasculature. Pulmonary arteries have a thin wall, relatively little smooth muscle, low activity, and high compliance. In adults, the normal pulmonary artery wall thickness is 40% ~70% of the normal aortic wall thickness in the same individual. The internal pulmonary arteries are generally classified as elastic pulmonary arteries, muscular pulmonary arteries, and pulmonary microvessels. Pulmonary arteries consist of three layers: the intima (a continuous layer of endothelial cells), the media (located between the inner and outer elastic membranes and consisting of SMCs, elastin, collagen, and proteoglycan), and the adventitia (composed of fibroblasts and loose collagen fibres). The outer diameter of elastic pulmonary arteries in adults is 500~1000 *μ*m, and the wall is composed of SMCs and abundant elastic fibres. The outer diameter of muscular pulmonary arteries is 50~ 100 *μ*m, and the inner and outer double layer elastic lamellae are composed mainly of SMCs. The average thickness of the muscle layer is 5% (3% ~ 7%). Pulmonary microvessels are small vessels in which the precursors of SMCs can differentiate into SMCs under pathological conditions, which makes arteries less than 80 *μ*m appear as double-layer elastic lamellae and an intact muscle layer. This pathological change has become recognized as an important cause of PH [10, 23].

Plexiform lesions are a typical histological feature of PAH. In addition, vasoconstriction, cell proliferation, and thrombosis are thought to be central to the pathogenesis of PAH. The early stage of PAH is histologically nonspecific; the only abnormality is membrane hypertrophy and mild thickening of the intima in the pulmonary artery, and characteristic plexiform lesions do not appear until the late stage. Plexiform lesions are arteriolar lesions at the distal end of the arterial branch (usually <300 *μ*m in diameter). There is much debate about whether plexiform lesions are a characteristic pathological change related to pulmonary vascular disease or markers of severe PH. However, pulmonary artery hypertrophy is generally observed, and excessive proliferation, reduced apoptosis, and PASMCs phenotypic switching play an important role in medial hypertrophy.

#### 2.1.3. PH Pathogenesis

Recent rapid developments in cell biology and molecular genetics have promoted further investigations of PH pathogenesis. It is currently believed that the occurrence of PH cannot be explained by a single pathophysiological mechanism but instead results from a combination of genetic, epigenetic, and environmental factors. The endothelial cells, SMCs, fibroblasts, and platelets are abnormally involved in its formation, and that a variety of vasoactive molecules, multiple ion channels, and multiple signalling pathways play an important regulatory role [24]. Many molecular mechanisms have been studied: aberrancies in the bone-forming protein type II receptor and activator receptor-like kinase genes; DNA damage, aberrancies in miRNAs; disruption of the proliferation/apoptosis balance, including endoplasmic reticulum stress, altered mitochondrial function, peroxidase proliferator activated receptor expression, elastase activity, calcium ion concentrations, and K^+^ ion channel activity; and abnormal vasoconstriction involving gas signalling molecules (NO, CO, and hydrogen sulfide), prostacyclin (PGI2), endothelin, and 5-hydroxytryptamine. Overall, the molecular mechanism is highly complex, involving a variety of signalling pathways.

### 2.2. Phenotypic Switching

The phenotype of vascular smooth muscle cells (VSMCs) is characterized by diversity and variability. During embryonic development, VSMCs gradually differentiate from an undifferentiated (synthetic) phenotype into a differentiated (contractile) phenotype with mature characteristics. However, when blood vessels are damaged or stimulated by various factors, VSMCs dedifferentiate from the contractile phenotype to the synthetic phenotype. This reversible shift in response to changes in environmental stimuli is called phenotypic switching [25].

#### 2.2.1. Characteristics and Marker Genes of Contractile VSMCs

In general, contractile VSMCs are smaller in size than synthetic VSMCs. Contractile VSMCs have an elongated, spindle-shaped morphology; are rich in myofilaments; express a large number of contractile-specific proteins; and have vital contractile ability. However, in these cells, DNA synthesis activity is low, and the ECM synthesis ability is poor; thus, their proliferation is very slow, and they do not migrate [26, 27]. Contractile VSMC marker genes include *α*-smooth muscle actin (*α*-SMA), smooth muscle myosin heavy chain (SMMHC), h1-calponin (CNh1), desmin, aortic carboxypeptidase-like protein (ACLP), metavinculin, telokin, h-caldesmon, smoothelin, and smooth muscle 22*α* (SM22*α*). These genes are usually upregulated in contractile VSMCs [27, 28]. SM22*α* has strict tissue specificity and cell phenotype specificity in smooth muscle tissue because it participates in remodelling of the actin cytoskeleton and regulates migration, contraction, and other behaviours. Caldesmon is a cytoskeletal protein that regulates cell contraction by interacting with myosin, actin, and calmodulin. It has two types, l-caldesmon and h-caldesmon, with the latter considered a specific marker for VSMC differentiation [29]. ACLP is an ECM-related secretory protein containing over 1100 types of amino acids; it can be produced by SMCs and contributes to VSMC proliferation and wound healing [30].

#### 2.2.2. Characteristics and Marker Genes of Synthetic VSMCs

**Figure 1:**
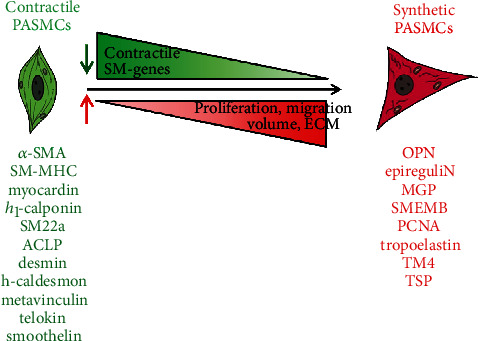
Characteristics and marker genes of contractile VSMCs and synthetic VSMCs.

Synthetic VSMCs have a rhomboid/epitheloid-like morphology with a large volume, few muscle filaments, and no contractility, but they can synthesize ECM (proteins), collagen, and osteopontin-8 (OPN-8) to simultaneously enhance cell proliferation and migration [31, 32]. Their marker genes include OPN, matrix gla protein (MGP), myosin heavy chain embryonic (SMemb), and tropomyosin 4 (TM4) [27, 28]. OPN, a secretory glycoprotein, is the most widely used synthetic marker protein and can regulate the phenotypic switching of VSMCs by activating multiple intracellular signalling-level interconnecting pathways, especially the mitogen-activated protein kinase (MAPK) pathway. Relevant studies have shown that the expression of *α*-SMA declines significantly and that of OPN increases significantly upon the conversion of contractile VSMCs into synthetic VSMCs [33]. The characteristics and marker genes of contractile VSMCs and synthetic VSMCs are summarized in Figure 1.

#### 2.2.3. Phenotypic Switching Mechanism

**Figure 2:**
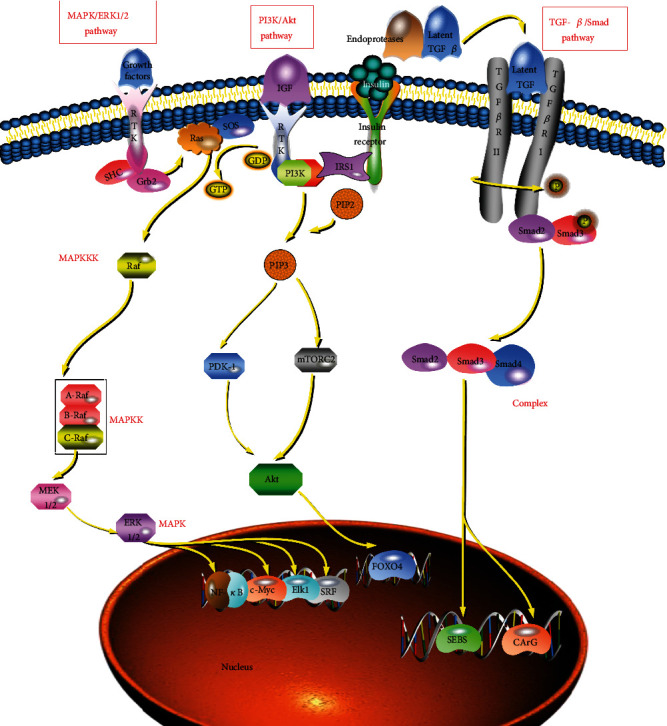
Phenotypic switching mechanism.

PASMCs phenotypic switching involves a variety of complex signal transduction pathways, mainly the MAPK/ERK1/2, phosphatidylinositol 3-kinase (PI3K)/Akt, and TGF-*β*/Smad pathways (see Figure 2). These signal transduction pathways regulate the differentiation direction of PASMCs by regulating the expression of SM-specific genes.

#### 2.2.4. MAPK/ERK1/2 Pathway

The signal transduction pathway represented by MAPK is called the MAPK pathway and is mainly composed of three central kinases: MAPK kinase kinase (MAPKKK), MAPK kinase (MAPKK), and MAPK. Four MAPK cascades have been identified, namely, the extracellular signal-regulated kinase (ERK), p38 MAPK, JNK, and ERK5 cascades [4].

Among these pathways, the MAPK/ERK1/2 pathway is the most well-known and widely studied and is closely related to cell proliferation and differentiation. When an extracellular ligand binds to a receptor tyrosine kinase (RTK) at the plasma membrane, signal transduction is initiated. The tyrosine residues on the receptor are then phosphorylated to form the Src homology 2 (SH2) binding sites. The adaptor protein Grb2, which contains SH2 domains, can bind with the receptor. Grb2 consists of one SH2 and two SH3 domains, which function to link upstream and downstream molecules. The two SH3 domains of Grb2 bind to proline-rich sequences in the son of sevenless (SOS) protein to activate the SOS. Next, activated SOS binds to Ras (an upstream activating protein), which promotes Ras to release guanosine diphosphate (GDP) and bind to guanosine triphosphate (GTP). Ras-GDP recruits Raf. Subsequently, Raf (acting as a MAPKKK) is activated. Once Raf is activated, any Raf family member (a-Raf, b-Raf, or c-Raf) can activate MEK1/2 (acting as a MAPKK). MEK1/2, in turn, activates ERK1/2 (acting as a MAPK). This sequence constitutes the important three-level Raf/MEK/ERK signalling cascade. Activated ERK1/2 can be translocated to the nucleus to activate ternary complex factors (TCFs) and other factors through phosphorylation. As a result, cells produce biological substances to respond to foreign signals [4, 34].

TCFs are ternary complexes formed by the binding of MYOCD and MYOCD-related transcription factor A/B (MRTF-A/B) with serum response factor (SRF) [35]. Among these components, MYOCD, an SMC-restricted transcriptional coactivator, is the most critical transcription factor discovered to date that inhibits the phenotypic switching of VSMCs. It can physically interact with SRF to selectively induce the expression of contractile marker genes such as SMA, SM22, and CNh1 to regulate switching to the contractile phenotype [36].

#### 2.2.5. PI3K/Akt Signalling Pathway

The PI3K/Akt pathway is one of the classical signalling pathways that regulates the phenotypic switching of VSMCs by regulating downstream transcription factors [37]. Insulin-like growth factor (IGF) and insulin signalling have been demonstrated to be able to inhibit VSMC dedifferentiation via the canonical PI3K/Akt pathway and maintain the contractile phenotype. Ligand-activated IGF or insulin receptors recruit insulin receptor substrates (IRS-1) and activate them by phosphorylation of the tyrosine residues. PI3K docking sites are subsequently formed, enabling PI3K to bind to its substrate, inositol phospholipids. Subsequently, PI3K converts phosphatidylinositol- (4,5-) bisphosphate (PIP2) into phosphatidylinositol- (3,4,5-) trisphosphate (PIP3). PIP3 provides docking sites for phosphoinositide-dependent kinase-1 (PDK1) and mTORC2. Akt is then activated by PDK1 (by phosphorylation of Thr308) and mTORC2 (by phosphorylation of Ser473). PDK1 can only partially activate Akt. However, mTORC2 can fully activate and phosphorylate additional substrates. Finally, activated Akt plays a role by phosphorylating downstream target proteins such as FOXO4. Phosphorylation of FOXO4, the substrate of Akt, inhibits phenotypic switching by promoting the nuclear export of FOXO4 and inhibiting MYOCD activity after Akt activation [31, 38].

#### 2.2.6. TGF-*β*/Smad Signalling Pathway

Transforming growth factor-*β* (TGF-*β*), a potent multifunctional soluble cytokine, exists in at least three isoforms: TGF-*β*1, TGF-*β*2, and TGF-*β*3. Its receptors are divided into two types, type I and type II, both of which are transmembrane serine/threonine receptors [39, 40]. TGF-*β* plays an important biological function in the phenotypic switching of mature SMCs. It promotes VSMC differentiation and maintains contractile phenotypes through both Smad-dependent and Smad-independent pathways [32]. Smad is a structurally related signal effector. In vertebrates, the genome encodes eight Smads—Smad1 to Smad8. Smad2 and Smad3 are mainly activated by TGF-*β* and the activin receptors T*β*RI and ActRIB, while Smad1, Smad5, and Smad8 are primarily activated by ALK-1, ALK-2, BMP-RIA/ALK-3, BMP-RIB/ALK-6, and other ligands.

In the Smad-dependent pathway, TGF-*β* is first activated by hydrolysis via endoproteinases. After TGF-*β* is activated, it binds to TGF-*β* II receptors. Next, TGF-*β* II receptors bind to TGF-*β* I receptors to form heterodimers. In these heterodimers, TGF-*β* II receptors can autonomously phosphorylate and activate TGF-*β* I receptors. Activated TGF-*β*1 receptors then recruit and activate Smad2 and Smad3. Subsequently, phosphorylated Smad2 and Smad3 form a complex with Smad4 and translocate to the nucleus to bind multiple Smad-binding elements and CArG and ultimately play related roles as transcription factors. Among the Smads, Smad3 is the primary mediator of TGF-*β* signalling; Smad3 can interact with SRF and MYOCD and activate the promoters of CArG-dependent VSMC genes. In the Smad-independent pathway, TGF-*β* can regulate VSMC phenotypic switching by activating the Erk, JNK, Notch, and p38 MAPK pathways [31, 39, 40].

### 2.3. PH and Phenotypic Switching

PH is a progressive pulmonary vascular disease characterized by five major features: vasoconstriction, cellular hyperplasia, high pulmonary arterial pressure, right ventricular heart hypertrophy, and vascular remodelling. It can be divided into five main groups. At present, the diagnosis and treatment of PH, especially PAH, are complex and challenging. Therefore, it is highly important to further reveal the potential molecular pathogenesis of PH and explore new therapeutic targets for PH [41, 42]. PH is a proliferative disease. As the understanding of this disease has increased, the phenotypic switching of SMCs from contractile to synthetic has attracted increasing attention. Dong et al. found that pulmonary vascular remodelling, as the core process in PH pathogenesis, is closely related to phenotypic switching [43]. Yeo et al. also noted that the PASMCs phenotypic switching induced by the loss of BMP signal transduction is an essential pathological basis of pulmonary vascular remodelling in PAH [44]. In addition, Morris et al. confirmed that VSMC phenotypes are strictly regulated by Notch3 and that abnormal Notch3 signalling plays a significant role in vascular remodelling [45]. Collectively, these results suggest that phenotypic switching plays an important role in PH. Therefore, further study of the mechanism underlying the occurrence and development of phenotypic switching is highly important for revealing the potential pathogenesis of PH.

## 3. Effect of miRNAs on PASMCs Phenotypic Switching in PH

### 3.1. miRNA Biogenesis and Mechanism of Action

RNA molecules in living organisms can be grouped into two categories, coding RNAs and noncoding RNAs, which constitute a highly complex RNA regulatory network in cells. Among RNAs, miRNAs are a class of single-stranded small noncoding RNAs that are evolutionarily highly conserved and are approximately 18–22 nt in length. miRNAs can directly degrade or repress the translation of their target mRNAs, thus negatively regulating gene expression at the posttranscriptional level.

miRNA biogenesis is a complex process. The most primitive form is the primary miRNA (pri-miRNA), which is approximately 300–1000 nt in length and is usually transcribed and synthesized by type II or type III RNA polymerase. Pri-miRNAs are first processed in the nucleus by Drosha RNase, which cleaves them into precursor miRNAs (pre-miRNAs) that contain approximately 70–90 nt and have a stem-loop structure [46]. Pre-miRNAs are transported from the nucleus to the cytoplasm via the Ran GTP-dependent transporter exportin-5 [47]. Via Dicer, a member of the RNase III family of nucleases that specifically cleaves double-stranded RNAs, pre-miRNAs are cleaved into double-stranded miRNA intermediates that contain approximately 22 nt and have a complementary double-helix structure. One strand is the mature miRNA, and the other strand is the miRNA∗ with the complementary sequence. Next, the double helix is unwound, and the mature miRNA strands are bound to the RNA-induced silencing complex (RISC) to form asymmetric RISC assembly [48]. This complex can bind to the target mRNA and cause its degradation or translational inhibition. The other strand (miRNA∗) is degraded immediately.

**Figure 3:**
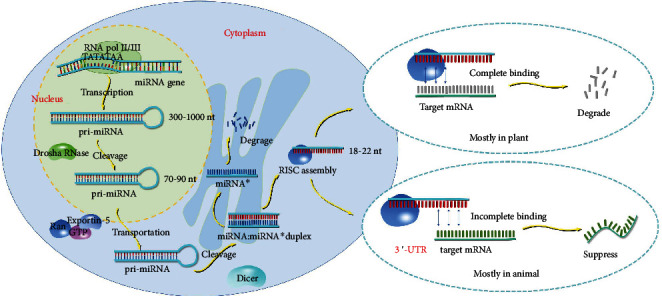
miRNA biogenesis and mechanism of action.

miRNAs can bind to their target mRNAs via two modes: complete binding and incomplete binding. In plants, miRNAs are almost completely paired with their target mRNAs and can degrade them by binding to multiple sites, including the coding region, of the target mRNAs [49]. In animals, the most common mode is incomplete complementary binding, which negatively regulates gene expression by miRNAs binding to the 3′-UTR of their target mRNAs. This binding mode generally does not affect the stability of the mRNA but can affect its translation. The biogenesis and mechanism of action are shown in Figure 3.

miRNAs are regulated by certain mechanisms. Only approximately 8% of human miRNAs are located in exons [50]. Intronic miRNAs are often regulated by their host genes and are processed from introns, but they may have distinct promoter regions, and their transcription is usually initiated by independent promoter elements. The transcription of miRNA genes can be initiated by upstream signal transduction and regulated by downstream transcription factors [51].

miRNA research has rapidly expanded in the several decades since the discovery of the first miRNA, Lin-4, in *C. elegans* by Lee et al. in 1993 and the subsequent discovery of the miRNA Let-7 [52, 53]. At least 30% of the genes in the human genome are estimated to be directly regulated by miRNAs [54]. Therefore, miRNAs are considered to be involved in almost all biological processes and play a pivotal role in various physiological processes, such as embryonic development, organogenesis, and tissue formation, as well as in many pathological processes, such as carcinogenesis, angiogenesis, and inflammation [55]. An increasing number of researchers have found that miRNAs play a unique and key role in the progression of PH by regulating PASMCs phenotypic switching, which is expected to be a potential target for the prevention of PH and related therapies [56]. Here, we review the research progress on miRNAs that regulate PASMCs phenotypic switching.

### 3.2. Roles of miRNAs in PH

miRNAs have been found to be widely involved in cardiovascular diseases such as hypertension. miRNAs are predicted to regulate various molecular mechanisms that are indispensable in the initiation, progression, and perhaps the attenuation or prevention of PH. However, the importance of only a few miRNAs in PH has been recognized. The strategy of combining system biology with traditional experimental approaches has recently contributed to the identification of novel miRNAs and their target genes/pathways, consequently raising awareness of the significance of miRNAs in PH.

The main miRNAs that have been discovered to promote the progression of PH include miR-17, miR-20, miR-27, miR-143/145, miR-210, and miR-221, while the main miRNAs that can delay and reverse the progression of PH include miR-34, miR-140–5p, miR-223, miR-451, miR-204, miR-424, and miR-503.

The classical mechanism of miRNA involvement in PH progression mainly affects pulmonary vascular remodelling by affecting cell proliferation, apoptosis, and phenotypic switching. For instance, an miR-140–5p mimic can affect the signal transduction of bone morphogenetic protein 4 (BMP4) and/or directly target the 3′-UTR of tumour necrosis factor-*α* [57, 58], inhibit the proliferation of PASMCs, and delay PH progression.

miRNAs can also inhibit the apoptosis of PASMCs. The expression of miR-34a-3p is decreased in PAH, which in turn upregulates the expression of mitochondrial dynamic protein (MiD) in PASMCs, accelerates mitosis, and reduces apoptosis [59]. miR-29b can inhibit the proliferation and induce the apoptosis of VSMCs by targeting the myeloid leukaemia 1 and cyclin D2 proteins [60].

In addition, the mechanism of miRNA involvement in the progression of PH includes affecting cell metabolism and inducing functional alterations in pulmonary vascular endothelial cells. Caruso et al. indicated that the overexpression of miR-124 or knockdown of polypyrimidine tract binding protein (PTBP1) can normalize the pyruvate kinase muscle isoform 2 (PKM2)/PKM1 ratio in pulmonary adventitial fibroblasts [61], reprogram mitochondrial metabolism, and reduce cell proliferation, which collectively alleviate PAH progression. Endothelin-1 (ET-1) is the most potent endogenous vasoconstrictor; it strongly regulates endothelial function and is a target gene of miR-98. Hypoxia can reduce the expression of miR-98 and increase the level of ET-1, thereby promoting the proliferation of pulmonary artery endothelial cells (PAECs) [62]. The expression of miR-29 family members, which are related to energy metabolism, was found to be decreased in PASMCs after exposure to the oestrogen metabolite 16*α*-hydroxyestrone, suggesting that miRNAs may also participate in the development of PH as hormone mediators [63].

In conclusion, miRNAs play an important and unique role in the development of PH through their complex regulatory network.

## 4. miRNAs Modulate PASMCs Phenotypic Switching

### 4.1. Growth Factor-Related miRNAs

Platelet-derived growth factor (PDGF) is a peptide growth factor that stimulates cell proliferation. It is currently considered to be the most potent growth factor that promotes the phenotypic switching of VSMCs. In PH, the amount of PDGF secreted by PAECs is significantly increased. PDGF treatment of PASMCs has been reported to significantly upregulate miR-221 [64], miR-15b [65], and miR-24 [66] and downregulate miR-21 [67], ultimately inducing cell phenotypic switching. Moreover, TGF-*β*/BMP signalling has been described as a negative regulator of the synthetic phenotype of VSMCs [32], and BMP stimulation can significantly downregulate the expression of miR-96 [68]. The mechanism by which PDGF regulates the expression of miRNAs may be related to antagonism of the BMP signalling pathway [67].

#### 4.1.1. Growth Factor-Related miRNAs That Promote Phenotypic Switching

*(1) miR-221*. The overexpression of miR-221 is associated with the proliferation of many types of tumours, such as breast and gastric cancer [69, 70]. It participates in vascular remodelling in AS by regulating angiogenesis activity and promoting the phenotypic switching of VSMCs [71]. In PH, miR-221 can promote PASMCs phenotypic switching by downregulating the target gene c-Kit. PH can cause extensive injury to small pulmonary vessels, resulting in the increased expression of PDGF ligands and receptors. Davis et al. showed that miR-221 is likely to be transcriptionally induced by PDGF signalling because they observed that both pri- and pre-miR-221 were significantly induced only 1.5 h after PDGF treatment [64]. miR-221 can directly target the 3′-UTR of the c-Kit and p27Kip1 mRNAs to mediate the differentiation and proliferation of PASMCs through distinct downstream mechanisms. Among these targets, p27Kip1 is downregulated at the translational level, which directly promotes cell proliferation. Four and a half LIM domain protein 2 (FHL2) is critical for the stabilization of MYOCD protein, which prevents MYOCD from degrading through the ubiquitin–proteasome-dependent degradation pathway [72]. The c-Kit signalling pathway may alter FHL2 gene expression or FHL2/MYOCD complex formation; these play roles in maintaining the stability of MYOCD and modulating SMC marker gene expression through direct transcriptional activation via the CArG box. PDGF treatment induces the expression of miR-221, leading to the downregulation of c-Kit mRNA expression, in turn inhibiting the transcription of SM-specific contractile genes by reducing the expression of MYOCD and thus playing a catalytic role in PASMCs phenotypic switching.

*(2) miR-15b*. The target genes of miR-15b mainly include proteins associated with cell proliferation (cyclin) [73], cell apoptosis (Bcl-2) [74], and cell invasion (NRP-2 and VEGFR-2) [75, 76]. miR-15b also participates in antiangiogenesis in the pathological process after myocardial infarction [77]. Kim and Kang showed that miR-15b is crucial for the PDGF-mediated inhibition of SM-specific genes [65]. miR-15b expression was found to be increased 1-fold within 4 h of treatment with PDGF, and the increased level of miR-15b was maintained for 24 h after PDGF stimulation, indicating that PDGF signalling regulates miR-15b expression. Previous studies have shown that miR-15b can promote the phenotypic switching of VSMCs. The inhibition of miR-15b expression under physiological conditions can promote *α*-SMA synthesis while maintaining the contractile phenotype of VSMCs [78]. That research group further found that miR-15b also mediates PASMCs phenotypic switching, but the specific mechanism remains unclear. Further investigations of predicted targets of miR-15b and the identification of the mechanisms underlying PDGF-mediated regulation of miR-15b will provide more evidence for elucidating the pathophysiological function of miR-15b in VSMC phenotypic regulation.

*(3) miR-96*. miR-96 plays a role in the proliferation and migration of multiple tumours, such as gastric cancer and ovarian cancer [79, 80], as well as in the maintenance of embryonic stem cell pluripotency. In addition, miR-96 can inhibit postinfarct neovascularization by targeting anillin. The downregulation of miR-96 expression can improve cardiac endothelial cell growth potential [81]. Kim et al. showed that the expression of pri-miR-96 was decreased in PASMCs under BMP4 stimulation in a Smad4-dependent manner [68]. In the pulmonary arteries, miR-96 negatively regulates the target gene Tribbles-like protein 3 (Trb3), resulting in a decrease in the expression of Smads, which in turn promotes PASMCs phenotypic switching. Trb3 is a BMPRII-interacting protein. BMP stimulation can release Trb3 from BMPRII; Trb3 then interacts with Smurf1 to degrade it through the ubiquitin–proteasome pathway. The reduction of Smurf1 stabilizes Smad proteins in the BMP pathway and consequently enhances the BMP reaction [82]. miR-96 is a critical molecule that functions as a negative regulator of the BMP signalling pathway and can thus inhibit SM-specific gene expression. The inhibition of BMP4 signalling may lead to upregulated miR-96 expression, further reducing the expression of Trb3 and resulting in a decrease in Smad protein expression, ultimately promoting the synthetic phenotype of VSMCs. Hence, there may be negative feedback regulation between miR-96 and Trb3 in PASMCs.

*(4) miR-24*. miR-24 is mainly involved in haematopoietic cell differentiation [83], tumour development, and other processes and widely participates in cardiomyocyte apoptosis, myocardial fibrosis, and cardiac remodelling after acute myocardial infarction [84, 85]. The miR-24 gene contains two members: miR-24-1 and miR-24-2. The expression level of the miR-24-2 cluster, but not the miR-2 cluster, was induced 1.5-fold after PDGF treatment for 4 h [66]. Similar to miR-96, miR-24 also targets Trb3 protein, causing a decrease in the expression of Smad proteins, such as Smad1 and Smad5, which inhibits the TGF*β* and BMP signalling pathways and consequently promotes the synthetic phenotype of PASMCs. The expression condition of miR-24 in PH patients needs to be further confirmed, which is hoped to provide a basis for the application of anti-miR-24 in PH treatment.

#### 4.1.2. Growth Factor-Related miRNAs That Inhibit Phenotypic Switching

*(1) miR-21*. miR-21 has long been valued for its role in tumour, cardiovascular, and lung diseases and is the most studied miRNA in PH vascular remodelling; however, the results of these studies have been contradictory. Some scholars who systematically studied miR-21 found that its expression is downregulated in the lung tissue of PH rats and in the lung tissue and plasma of idiopathic PAH (IPAH) patients. In PASMCs, PDGF significantly downregulates miR-21 expression [67, 86].

In the maintenance of the contractile phenotype in VSMCs, the BMP4 signalling pathway has always been considered to be important. Aberrant expression or inactivating mutations in the BMP receptor (BMPR) gene can lead to VSMC dedifferentiation.

Studies have shown that miRNAs play an important role in the BMP-mediated promotion of the VSMC contraction phenotype, and miR-21 is one example [78]. Upon BMP treatment, R-Smad proteins associate with pri-miR-21 in the complex with Drosha to promote the processing of pri-miR-21 to pre-miR-21 and increase miR-21 levels [78, 87]. By inducing miR-21 expression, BMP4 leads to the downregulation of programmed cell death 4 (PDCD4), which inhibits contractile gene expression.

In addition, Kang et al. found that in PASMCs almost all dedicator of cytokinesis (DOCK) family members are miR-21 targets [67]. BMP4-mediated upregulation of miR-21 promotes the maintenance of the PASMCs contractile phenotype and inhibits PASMCs migration by inhibiting DOCK4, -5, and -7. In addition, PDGF stimulation can promote PASMCs phenotypic switching through the miR-21/DOCK signalling pathway.

miR-21 has been identified as a biomarker of some tumours, and regulating its function is a cardioprotective strategy. Whether it is also involved in right heart failure in PH is worth further investigation.

*(2) miR-132*. miR-132 is believed to play an important role in the central nervous system and cardiovascular system. Some researchers have found that miR-132 expression is upregulated in myocardial hypertrophy, hypertension, AS, and other diseases [88–90], which suggest a strong effect of miR-132. This is closely related to its influence on the proliferation and migration of endothelial cells and VSMCs. One study found that miR-132 was upregulated in monocrotaline- (MCT-) induced PH rats and PDGF-induced PASMCs [91], and further studies found that miR-132 has a complex role in the occurrence of PH, inhibiting PASMCs proliferation and maintaining the PASMCs contraction phenotype while promoting cell migration, which was achieved by targeting phosphatase and tensor protein homology (PTEN). The antiproliferative and phenotypic switching-inhibiting effects of miR-132 do not appear to coincide with the increase in miR-132 in MCT-induced PH rats. The cause for this may be multifaceted, and possibly PASMC proliferation is induced by other factors. Based on these results, whether inhibiting miR-132 can prevent and treat PH indeed needs further study.

### 4.2. Hypoxia-Related miRNAs

The mechanism by which hypoxia leads to changes in miRNA expression can be mediated by hypoxia inducible factor-1- (HIF-1-) dependent and HIF-1-independent pathways. Although hypoxia reportedly induces PASMCs phenotypic switching, the expression levels of miR-23a [92], miR-9 [93], miR-214 [36], and miR-20a [94] are increased, while those of miR-449 [95], miR-206 [96], miR-124 [97], miR-30c [98], and miR-140 [99] are decreased.

#### 4.2.1. Hypoxia-Related miRNAs That Promote Phenotypic Switching

*(1) HIF-1α-Dependent Pathway*. HIF-1 is an important mediator of oxygen homeostasis and is a nuclear transcription factor that plays an active role in hypoxia. HIF-1 is a heterodimer composed of an oxygen-sensitive *α* subunit (HIF-1*α*) and a constitutively expressed *β* subunit (HIF-1*β*). Under normoxic conditions, HIF-1*β* is stably expressed in the cytoplasm, while HIF-1*α* is promptly degraded by the hydrolytic ubiquitin protease complex after its translation. In contrast, the protein level of HIF-1*α* increases rapidly due to the inhibition of HIF-1*α* degradation under hypoxic conditions. The physiological activity of HIF-1 mainly depends on the function and expression of the *α* subunit; thus, HIF-1*α* is called the active subunit of HIF-1 [100]. Many studies have confirmed that HIF-1 is upregulated and plays an important role in various types of PH, especially in hypoxic PH (HPH) [101]. In the early stage of hypoxia, HIF can activate the transcription of more than 100 genes, including miRNAs [92, 93], which affect and regulate various pulmonary vascular functions, such as reactive oxygen species generation, angiogenesis, and vascular homeostasis, including PASMCs phenotypic switching.

*miR-23a:* miR-23a is involved in the development of various cancers, promotes cardiac hypertrophy, and antagonizes muscle atrophy [102, 103]. Yan et al. showed that under hypoxic conditions the expression of miR-23a was upregulated in primary rat PASMCs through a mechanism involving HIF-1a [92]. In addition, the expression of contractile protein markers was significantly downregulated, suggesting that miR-23a regulates the dedifferentiation of PASMCs. Moreover, the level of contractile protein markers was significantly downregulated after transfection of a miR-23a mimic for 48 h under normoxic conditions, while the expression of contractile protein markers in miR-23a inhibitor-transfected cells was significantly increased under hypoxic conditions. HIF-1*α* was transcriptionally activated in PASMCs cultured under hypoxic conditions and participated in the transcriptional activation of miR-23a by binding to the regulatory element upstream of their transcription start sites (TSS), in turn downregulating the expression of contractile phenotype marker proteins in PASMCs, an effect that may be related to the enhancement of cell phenotypic switching. The downstream targets of miR-23a in the inhibition of contractile protein marker expression have not been identified.

*miR-9:* miR-9 functions in promoting or antagonizing proliferation according to cell type specificity. For instance, miR-9 promotes the proliferation of gastric carcinoma cells by targeting caudal type homeobox 2 [104, 105], while it inhibits the proliferation of nasopharyngeal, breast, and ovarian carcinoma [106–108]; reduces macrophage foam cell formation; and participates in the regulation of the AS process by targeting the human ACAT1 gene [109]. miR-9 may directly inhibit the transcription of genes encoding SM-specific proteins by targeting MYOCD, thereby promoting phenotypic switching. Shan et al. found that miR-9 was increased in primary rat PASMCs exposed to hypoxia via the activation of HIF-1*α* [93]. HIF-1 binding motifs (5′-RCGTG-3′) are located in the region within 5 kb upstream of the TSS at miR-9 loci, and HIF-1*α* enrichment was increased at all HIF-1 binding motifs upstream of the miR-9 TSS after 24 and 48 h of hypoxia exposure.

*(2) HIF-1ɑ-Independent Pathway*. Similar to miR-9, both miR-214 and miR-20a are upregulated in HPH and promote PASMCs phenotypic switching through negative regulation of the MYOCD signalling pathway [36, 94]. However, they regulate MYOCD through different mechanisms.

*miR-214:* miR-214 participates mainly in cancer and is an important regulator of fibrosis in the liver, kidney, and myocardium. It plays a regulatory role in promoting hypertrophy in myocardial hypertrophy and heart failure [110]. miR-214 directly inhibits the MYCOD-LMOD1 signalling pathway and promotes PASMCs phenotypic switching by inhibiting the downstream target myocyte enhancer factor 2C (MEF2C) [36]. Sahoo et al. found that miR-214 was upregulated in PASMCs isolated from PAH patients and hypoxia-induced human PASMCs (hPASMCs), while leiomodin1 (LMOD1), MYOCD, and MEF2C were downregulated [36]. MEF2C serves as an upstream coordinator of SMC differentiation and cooperates with MYOCD to regulate the SMC contractile phenotype [111]. LMOD1 is an SM-specific gene regulated by MYOCD at the transcriptional level and may play an important role in SMC contractile activity and actin cytoskeleton assembly through its association with tropomyosin during smooth muscle cell contraction [112]. Research has shown that MEF2C and LMOD1 are direct targets of miR-214. miR-214 directly regulates the expression of SM-specific genes at the level of LMOD1 and through the upstream disruption of the MEF2C/MYOCD pathway. Exogenous administration of a miR-214 inhibitor to hPASMCs of PH patients restored the contractile phenotype of these cells, suggesting that miR-214 plays a key role in promoting the occurrence and development of PH.

*miR-20a:* the dysregulation of miR-20a expression is involved in the development of a variety of cancers [113]. In PH, miR-20a targets PKG1, promotes the activation of Elk-1, and competes for the binding sites of MYOCD and SRF, eventually leading to the dissociation of the MYOCD-SRF complex and termination of the SM-specific gene expression programme. Zeng et al. found that miR-20a was gradually increased with prolonged exposure to hypoxia in HPH mice and in hypoxic hPASMCs [94]. Unlike miRNAs that bind to the 3′-UTR sequence of the target mRNA, miR-20a may regulate the expression of the PKG1 gene by binding to the coding region of PKG1. PKG is a serine/threonine-specific protein kinase that phosphorylates its substrate proteins to achieve signal transduction. Studies have confirmed that PKG can regulate the dedifferentiation of SMCs by promoting the expression of MYOCD, inhibiting the expression of SRF and Elk-1, and inhibiting their binding to CArG elements in SM-specific genes.

#### 4.2.2. Hypoxia-Related miRNAs That Inhibit Phenotypic Switching

miR-449 [95], miR-206 [96], miR-124 [97], miR-30c [98], and miR-140 [99] are downregulated in PASMCs and in the lung vasculature of animal models of HPH and inhibit PASMCs phenotypic switching by acting on their respective targets.

*(1) miR-449*. miR-449 clusters, located in cancer susceptibility sites, inhibit tumour growth, invasion, and metastasis by acting on multiple signalling factors (including the Notch pathway, VEGF, and P53) and promoting apoptosis and differentiation. The miR-449a/c-Myc axis reportedly plays an important role in regulating PASMCs phenotypic switching and pulmonary vascular remodelling [95]. miR-449 was downregulated in rat PASMCs in hypoxia-induced PH and directly targeted c-Myc regulation to promote the expression of SM-22*α*, calponin, and myosin, which ultimately inhibited PASMCs phenotypic switching. It is known that c-Myc expression is inhibited in resting and terminally differentiated cells, while it is temporarily activated in the initial stage of proliferation, after which it peaks rapidly and then recovers to baseline [114]. Hypoxia-induced downregulation of SM-specific genes and upregulation of OPN were significantly reversed by c-Myc knockdown. Moreover, miR-449 was found to regulate mitochondrial function in PASMCs by targeting c-Myc [95].

*(2) miR-206*. miR-206 is abnormally expressed in gastric cancer, breast cancer, liver cancer, and lung cancer and is a metastatic suppressor for many cancers. Studies have found that miR-206 targets the Notch3 gene to regulate skeletal muscle cell proliferation and cell cycle block [115]. The expression of miR-206 was significantly decreased in PASMCs from hypoxia-induced PH mice compared to control mice. The overexpression of miR-206 in hPASMCs promoted apoptosis and inhibited proliferation. Moreover, compared with the control hPASMCs, *α*-SM actin and calponin were higher in hPASMCs overexpressing miR-206, while miR-206 downregulation decreased the expression of these proteins in hPASMCs. These findings support an important role for miR-206-mediated signalling in maintaining the differentiation phenotype of hPASMCs. Additionally, miR-206 was found to play a role by inhibiting Notch3 signalling [96].

Notch3 signalling significantly affects the development of PAH [116]. It can influence the stability of blood vessels because of the interaction between the Notch3 target gene and the BMPR target gene. Notch3 is overexpressed in VSMCs in PAH [116], but the cause of this steady-state increase is unknown. However, miR-206 downregulation in PH may be a reason for the increase in Notch3 expression in PH [96]. miR-206 can significantly increase the expression of SM-specific proteins (*α*-SMA and calponin) in PASMCs by inhibiting the corresponding pathways. Correcting altered expression of miRNAs could be a potential therapeutic strategy for PAH.

*(3) miR-124*. miR-124 is rich in the brain, with high expression in normal tissues but low expression in many cancers (such as colorectal cancer, breast cancer, gastric cancer, and pancreatic cancer). miR-124 expression level was also lowered in diseases such as Parkinson's disease, Huntington's disease, hypertension, and PH [117]. Upregulating miRNA-124 in vivo can delay these disease processes. miR-124 may play a role in promoting contractility and maintaining the differentiated phenotype of PASMCs by suppressing the nuclear factor of activated T cell (NFAT) pathway [97]. Studies have found that NFAT signalling is associated with PASMCs proliferation and PAH. One study showed that NFATc2 was upregulated and activated in PAH patients [118]. Additionally, the expression and activation of NFATc3 were increased in a hypoxia-induced PH mouse model, and the proliferation and migration of PASMCs are also regulated by the CAN/NFAT signalling pathway [119, 120]. It was recently reported that ectopic overexpression of three different NFAT isoforms significantly downregulates *α*-SMA expression, indicating its role in PASMCs phenotypic switching. These results demonstrated that the NFAT-mediated signalling pathway plays a major role in the pathogenesis of PH. miR-124 inhibited NFAT signalling by suppressing both the activation and nuclear translocation of NFAT by targeting numerous genes, including NFATc1, calmodulin-binding transcription activator 1, and PTBP1 [97].

*(4) miR-30c*. miR-30c expression is reduced in various human tumour tissues, and some studies have identified it as a potential biomarker of prostate cancer, bladder cancer, and breast cancer. There are development prospects for miR-30c in the diagnosis and treatment of partial remissions. Studies have shown that hypoxia inhibits miR-30c expression in PASMCs, resulting in the proliferation of PASMCs and inhibition of their apoptosis. Moreover, a miR-30c inhibitor directly promotes PASMCs phenotypic switching from contractile to synthetic, while miR-30c mimic treatment under hypoxic conditions could reverse these effects by inhibiting the PDGF signalling pathway by targeting PDGF receptor *β* (PDGFR-*β*) [98]. PDGFR-*β* plays a crucial role in the regulation of cell function. Accumulating evidence suggests that abnormalities in the PDGF/PDGFR signalling pathway are involved in PH pathogenesis [121]. In various experimental models and in humans, the upregulated expression of PDGF and PDGFRs was associated with PH [122–124]. Interestingly, miR-30c-mediated changes in PDGFR expression were found to occur only in hypoxic cells, not in PDGF-stimulated cells. In addition, miR-30c can participate in ventricular remodelling by targeting XBP1, TGF-1, and others [125], and whether it is involved in right ventricular remodelling in PH is worth exploring.

*(5) miR-140*. miR-140 was found to play an important role in breast cancer [126], non-small-cell lung cancer, and osteoarthritis [127, 128]. It can also be used as a predictor of coronary heart disease. miR-140 expression was found to be significantly downregulated in lung tissues of congenital PH and PAH patients [99]. Compared with patients without PH, patients with congenital PH have higher pulmonary artery pressure and lower miR-140 expression. Correlation analysis showed that miR-140 expression was negatively correlated with pulmonary artery pressure and the expression of Wnt signalling pathway-related proteins (Wnt-1 and *β*-catenin). Specifically, under hypoxic conditions, miR-140 expression was downregulated in hPASMCs, while Wnt-1 expression was upregulated. The upregulation of miR-140 increased the expression of SM-specific proteins; for example, it significantly increased *α*-SMA, SM22, and calponin expression. Transfection with an miR-140 inhibitor led to an increased cell proliferation rate, increased migration, and reduced expression of contractile phenotype-related proteins, suggesting that miR-140 is necessary for maintaining the PASMCs phenotype. Further research showed that the Wnt-1 3′-UTR contains miR-140 recognition sites, suggesting that miR-140 directly targets Wnt-1 and suppresses PASMCs phenotypic switching. In addition to Wnt signalling, miR-140 also directly targets Dnmt1, decreases SOD2 expression, and inhibits phenotypic switching of hPASMCs [99]. Moreover, one study showed that delivering miR-140 into the lungs with liposomes reduced haemodynamic indicators and pulmonary vascular reconstruction in rats by suppressing Smurf11 expression [129]. The delivery of miR-140 to the lungs using liposomes may offer new possibilities for treating PH.

*(6) miR-17~92*. In the human genome, the miR-17~92 cluster, a typical polycistronic miRNA gene cluster, is located on chromosome 13. This cluster encodes and expresses six mature miRNAs, namely, miR-17, miR-20a, miR-18a, miR-19a, miR-19b, and miR-92a, each of which has its own target gene and biological function. It regulates tumour angiogenesis and widely participates in the development of lymphocytes [130]. The expression imbalance of this cluster can lead to a variety of diseases, including haematologic neoplasms, solid neoplasms, immune diseases, and cardiovascular diseases [131].

miR-19a/b and miR-17/20a are important members of the miR-17~92 cluster. The expression of miR-19a/b and miR-17/20a was downregulated in PASMCs of patients with IPAH and PAH associated with other diseases (APAH). Interestingly, in mice with HPH, miR-17~92 was found to be upregulated in the early stage of HPH but downregulated in the later stage [132]. Specific knockout of miR-17~92 in VSMCs attenuated HPH progression and pulmonary artery pressure in mice, but the effect of miR-17~92 knockout was counteracted by the addition of recombinant miR-17~92, suggesting that miR-17~92 cluster members strongly participate in pulmonary vascular remodelling in HPH. Interestingly, miR-17~92 was found to promote both proliferation and differentiation of PASMCs in vitro. Its mechanism regulating the phenotypic switching of PASMCs is independent of the MYOCD pathway but depends on the TGF-*β* signal. These results suggest that miR-17~92 in PASMCs is an important participant in the pathogenesis of PH but not the absolute controller. In PASMCs, the direct downstream targets of miR-17~92 have been confirmed to include plasminogen activator inhibitor 1 (PAI-1), PDZ, LIM domain 5 (PDLIM5), and prolyl hydroxylase domain-containing 2 (PHD2).

Among the miR-17~92 cluster members, miR-19a/b can positively regulate the TGF-*β*/Smad2/calponin signalling pathway by inhibiting PAI-1 in PASMCs to maintain the contraction phenotype [132]. PAI-1 can be secreted by PASMCs and act as a major inhibitor of tissue-type and urokinase-type plasminogen activators, and it mainly regulates the plasma fibrinolysis system and cell adhesion ability. Studies show that suppression of PAI-1 can increase Smad2 and the expression of SMC markers, thus negatively regulating the PASMCs contractile phenotype and influencing its metabolism [132]. Notably, PAI-1 was not abnormally expressed in APAH and IPAH PASMCs but was downregulated in the rat HPH model, suggesting that this pathway is regulated differently in different groups of PH.

miR-17/20a is another important member of the miR-17~92 cluster, and it can inhibit PASMCs phenotypic switching by inhibiting PDLIM5 and promoting the TGF-*β*3/Smad3 signalling pathway [133]. PDLIM5 acts as an adaptor protein to sequester transcription factors in the cytoplasm; it can also interact with kinases to exert its effects and can inhibit the expression of SMC markers through TGF-*β*3/smad3 signal transduction. Therefore, it serves as a negative regulator of the SMC contractile phenotype. miR-17/20a can directly target PDLIM5 and decrease its expression. In addition, miR-17/20a can indirectly inhibit PAI-1 by regulating PDLIM5. Further studies have shown that miR-17~92 also upregulates the expression of HIF-1*α* by inhibiting PHD2 in PH [134], promoting the proliferation of PASMCs and enhancing pulmonary vessel remodelling. Therefore, the different effects of miR-17~92 may be closely related to the biphasic regulation of early upregulation and late downregulation during PH progression.

In summary, the miR-17~92 cluster plays a complex regulatory role in the development of PH. The regulation of PAH pathogenesis by the miR-17~92 cluster requires further study and may become a potential therapeutic direction for PH.

*(7) miR-let-7g*. miR-let-7 is widely expressed in the cardiovascular system, with abnormalities in many cardiovascular diseases, such as myocardial hypertrophy, myocardial fibrosis, myocardial infarction, angiogenesis, AS, and hypertension. Our team used miRNA network pharmacology to reveal that the let-7 family was involved in three functional pathways: TGF-/BMP, hypoxia, and inflammation. We also found that miR-let-7g was decreased in hypoxic PH rats. It can inhibit hypoxia-induced PASMCs proliferation by targeting c-Myc [135]. Considering that miR-449 can inhibit the phenotypic switching of PASMCs by targeting c-Myc [95], it is also worth exploring whether let-7g can also inhibit the phenotypic switching of PASMCs by targeting c-Myc. We also found that let-7g can negatively regulate LOX-1 expression in PH [136], and LOX-1 can promote the phenotypic switching of PASMCs through the ERK1/2-Elk-1/MRTF-A-SRF signalling pathway [25]. Additionally, it is known that after activation of the MAPK pathway, ternary complex factor (TCF) is activated and can promote the phenotypic switching of VSMCs by remodelling chromatin containing the CArG box promoter. This process isolates SRF and reduces troponin expression. Using bioinformatics prediction, our team found that let-7g targets MEKK1 to regulate the MAPK pathway. Based on the above findings, we conjecture that let-7g can likely inhibit PASMCs phenotypic switching, and this is one of our future research directions.

## 5. Conclusions and Future Directions

**Figure 4:**
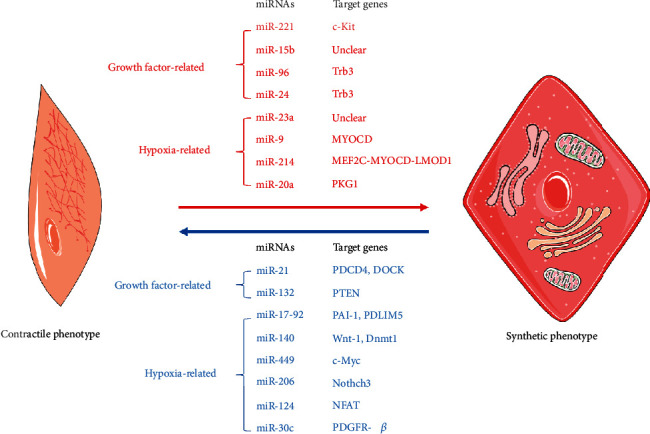
miRNAs that influence PASMC phenotypic switching.

PH is a serious progressive cardiopulmonary disease with poor prognosis and no effective treatment. Researches worldwide have committed to exploring its pathogenesis and identifying new targets for its prevention and treatment. In recent decades, international research on miRNAs has rapidly progressed, and the discovery of miRNAs has opened a new research area to investigate mechanisms of disease occurrence and development. miRNAs play important roles in various biological systems and diseases. Recent studies have demonstrated that miRNAs can regulate many key molecular pathways that play crucial roles in the occurrence, development, and, possibly, the attenuation or prevention of PH. These miRNAs can be classified into growth factor-related miRNAs, inflammation-related miRNAs, and hypoxia-related miRNAs based on the affected signalling pathways [137]. Compared with research in other fields, research on miRNAs in the occurrence and development of PH is relatively underdeveloped. Only a few miRNAs have been proven to be suitable therapeutic targets for this disease, and almost no research has been conducted to investigate the effects of miRNAs on ion channels in PAECs and PASMCs. However, this deficit also presents a rare opportunity for us. Enhancing the study of PH-related miRNAs will help to further clarify the pathogenesis of PH, develop more effective targeted drugs, and assist in the early diagnosis. At present, clinical treatment can only reduce symptoms and cannot reverse the disease process. Although the majority of miRNA research and miRNA-based therapeutics in current clinical trials are related to cancer, they still have great prospects in the treatment of pulmonary diseases [138]. Some animal experiments have proven that drugs targeting miRNAs can delay or even reverse the PH process. For example, in a rat PH model induced by monocrotaline, an airway atomized miR-140 simulant and miR-223 simulant were shown to treat PH [57, 139]. Therefore, miRNA-based therapeutics have become a new hope for the reversal of PH symptomatology. However, further research is needed to elucidate how to accurately deliver miRNAs into lung tissue while avoiding adverse reactions in other tissues and how to maintain stability in cells. Simultaneously, the role of miRNAs in vivo is complex, and there may be multiple targets that form a network with each other. From this perspective, miRNAs that have little impact on other organs but have obvious effects in PH may be more valuable for research. Furthermore, analysing PH-related miRNAs and studying their upstream and downstream targets would also be helpful in exploring the aetiology and laying a foundation for finding new powerful targets and developing related, non-miRNA drugs. This review summarizes the role of miRNAs in pulmonary vascular remodelling in PH via the regulation of phenotypic switching and summarizes the potential target genes (see Figure 4). Researchers are expected to focus on PASMCs phenotypic switching to reveal new roles of miRNAs in PH occurrence and development. Recent reports have shown that extracellular miRNAs bind to protein complexes and are thus not readily degraded by RNases in the circulation; in addition, they exhibit stable and abundant expression and can thus be used as potential biomarkers for the diagnosis or early detection of PH [140]. miRNA molecules have a simple structure and low molecular weight and are easy to synthesize and modify; furthermore, miRNA-based treatments can not only silence genes (and eliminate undesirable protein translation) but also restore the expression of lost proteins to physiological levels. For these reasons, drugs targeting miRNAs are expected to constitute a new generation of molecular approaches for PH treatment.
